# The clinical significance of myeloid-derived suppressor cells in dengue fever patients

**DOI:** 10.1186/s12879-019-4574-2

**Published:** 2019-11-01

**Authors:** Peng-Le Guo, Ling-Hua Li, Wen-Li Li, Jin-Cun Zhao, Feng-Yu Hu, Fu-Chun Zhang, Wei-Ping Cai, Xiao-Ping Tang

**Affiliations:** 1 Department of Infectious Diseases, Guangzhou Eighth People’s Hospital, Guangzhou Medical University, Guangzhou, 510060 Guangdong Province China; 2 Department of Infectious Diseases, Guangdong Second People’s Hospital, Guangzhou, 510317 Guangdong Province China; 3 Guangzhou Institute of Respiratory Disease, the First Affiliated Hospital, Guangzhou Medical University, Guangzhou, 510120 Guangdong Province China

**Keywords:** Myeloid-derived suppressor cells, Dengue fever, Inflammatory storm

## Abstract

**Background:**

Myeloid-derived suppressor cells (MDSCs) play immunosuppressive roles in cancers and some infectious diseases; however, their role in dengue fever (DF) remains unknown. This study evaluated the clinical significance of MDSCs in DF patients.

**Methods:**

This study comprised 178 non-severe DF patients, 20 non-dengue fever (NDF) controls, and 30 healthy donors. The DF patients were divided into the following five groups based on the fever duration from its onset to the day of sample collection: fever duration of 1–2, 3–4, 5–6, 7–8, and > 9 days. Among these DF patients, 14 were monitored for eight days, and their peripheral blood samples were collected every two days. The mononuclear cells were isolated and analyzed using flow cytometry. The correlation between the MDSCs and clinical and immunological indicators of the DF patients was evaluated using Spearman analysis.

**Results:**

The count of the peripheral blood MDSCs, especially monocytic MDSCs, of the 178 DF patients were dramatically higher than those of the NDF and healthy controls, and remarkably decreased with the fever duration. Moreover, the MDSC count correlated with some indicators, including the dengue viral load (*rho* = 0.367, *p* < .001), body temperature (*rho* = 0.263, *p* = .005), prothrombin time (*rho* = 0.475, *p* < .001), CD4^+^ T cell number (*rho* = − 0.317, *p* < .001), CD8^+^ T cell number (*rho* = − 0.361, *p* < .001), “programmed cell death protein 1” (PD-1) (*rho* = − 0.347, *p* < .001), “T cell immunoglobulin domain and mucin domain-3” (Tim3) (*rho* = − 0.258, *p* = .001), interferon-α (IFN-α) (*rho* = 0.43, *p* < .001), and “regulated upon activation normal T-cell expressed and secreted” (RANTES) (*rho* = 0.278, *p* = .019). Furthermore, the level of arginase-1, but not nitric oxide, was higher in the DF patients than in the healthy controls and was closely related to the number of MDSCs (*rho* = 0.265, *p* = .024).

**Conclusions:**

Our study reveals a significant correlation between MDSCs and DF clinical indicators, posing MDSCs as potential target cells for DF treatment.

## Background

Dengue fever (DF), an arbovirus-caused disease, is one of the most threatening viral diseases caused by dengue virus (DENV) in humans and has a wide distribution and high incidence rate worldwide. The outbreak of dengue fever in Guangdong province in 2014 was the most serious one during the past 20 years. The number of patients with DF exceeded 45,000 [1]. In the following years, the disease spread to more new areas in China and has been a major challenge since then. Due to the lack of ideal animal models, the pathogenesis of DF remains unclear. However, it has increasingly been proven that the host immune-related mechanism is dramatically important for the disease progression of DF. There is some evidence suggesting that soluble pro-inflammatory cytokines, also known as cytokine storm, may play an important role in the pathological process [2, 3]. Therefore, understanding the mechanisms that lead to the pathological damage of DENV infection may be useful for developing novel immunotherapies against DF.

Myeloid-derived suppressor cells (MDSCs) are a group of immature cells derived from bone marrow and are prevented from full differentiation under certain pathological conditions such as cancer, inflammatory diseases, and autoimmune disorders [4–8]. In the mouse models they have Gr1 + CD11b + phenotype [4], while in the human body they usually have HLA-DR−/lowCD33 + CD11b + phenotype in humans [5]. To date, MDSCs have mostly been studied from an oncology perspective. However, recent studies have found that the proportion of MDSCs increases in many viral infectious diseases, such as influenza A, viral hepatitis, and acquired immune deficiency syndrome (AIDS) through inhibiting T-cell immune response and participating the course of disease progression [9–11]. Although MDSC expansion is a common response to inflammatory processes, the level of MDSCs in DF patients and its role in the disease progression remain unclear. In this study, we aimed to investigate the clinical significance of MDSCs in DF patients by evaluating the correlation between MDSCs and DF disease indicators.

## Methods

### Study participants

The patients and controls were recruited between June and September 2014 from Guangzhou Eighth People’s Hospital, China, which is the largest hospital specialized in infectious diseases in Southern China. 178 non-severe DF patients who met the following diagnostic criteria of DF according to the Guidelines for Diagnosis and Treatment of Dengue in China, 2014 were recruited for the study [12]: 1) epidemiological history; 2) clinical findings including fever, nausea, vomiting, headache, arthralgia, retro-orbital pain, rash, myalgia, and hemorrhagic manifestations or leucopenia, etc.; 3) at least one positive test result for dengue [detection of the nonstructural protein 1 (NS1) or reverse transcriptionpolymerase chain reaction (RT-PCR), or > 4-fold increase in the titer of the serum-specific IgG antibody during the recovery period]. Patients who met the following criteria were excluded: 1) severe DF with hemorrhagic evidence, shock and severe organ damage; 2) Long-term use of hormones and immunosuppressive agents. In total, 178 DF patients were recruited, which were then divided into the following 5 groups based on the fever duration from the fever onset until the samples were collected: fever duration of 1–2 (Group A), 3–4 (Group B), 5–6 (Group C), 7–8 (Group D), and > 9 days (Group E). Group A contained 14 patients who were followed up for eight days, and their blood samples were collected every two days. Additionally, 20 patients with fever but negative test result(s) for dengue [non-dengue fever (NDF) controls] and 30 healthy donors were recruited from the same hospital clinic. The inclusion time of the participants in both NDF and the healthy control groups was the same as that in DF groups. The overall basic characteristics of the DF patients and their distribution among the 5 groups are outlined in Table 1 and the overall basic characteristics of the 14 follow-up DF patients are listed in Table 2.

**Table 1:** Basic characteristics of the 178 DF patients (Groups A–E)

Variables	Total	Group A	Group B	Group C	Group D	Group E
No. of patients	178	40	42	32	33	31
men/woman	99/79	18/22	19/23	13/19	15/18	14/17
Age (yr) [range (avg)]	(18–55) 25	(20–55) 27	(18–53) 24	(18–55) 26	(18–50) 24	(18–55) 29
Serum dengue RNA median IU/m (range)	3 × 10^7^ (27.6–4 × 10^8^)	5 × 10^7^ (3 × 10^7^–4 × 10^8^)	5 × 10^7^ [(1.9–8.9) × 10^7^]	6.7 × 10^6^ (4.76 × 10^6^–1.8 × 10^7^)	7.2 × 10^4^ (1.4 × 10^3^–1.5 × 10^5^)	2.4 × 10^3^ (27.6–4.5 × 10^3^)
ALT median U/L (range)	75 (13–582)	24 (13–28)	49 (31–65)	77 (35–118)	84 (57–111)	111 (62–582)
Temperature median °C (range)	37.5 (36–40)	38.5 (37.5–40.0)	37.9 (37.1–38.7)	37.4 (37.0–37.8)	36.9 (36.6–37.2)	36.5 (36.0–36.6)
WBC median/L (range)	3.5 × 10^9^ (1 × 10^9^–7.9 × 10^9^)	4.5 × 10^9^ (3.9 × 10^9^–7.9 × 10^9^)	2.4 × 10^9^ (1.0 × 10^9^–2.7 × 10^9^)	2.5 × 10^9^ (2.2 × 10^9^–2.9 × 10^9^)	3.4 × 10^9^ (2.8 × 10^9^–4.0 × 10^9^)	4.5 × 10^9^ (4.1 × 10^9^–5.0 × 10^9^)
PLT median/L (range)	137 × 10^9^ (83 × 10^9^–377 × 10^9^)	144 × 10^9^ (127 × 10^9^–160 × 10^9^)	106 × 10^9^ (90 × 10^9^–121 × 10^9^)	102 × 10^9^ (83 × 10^9^–120 × 10^9^)	124 × 10^9^ (91 × 10^9^–155 × 10^9^)	215 × 10^9^ (181 × 10^9^–373 × 10^9^)
Prothrombin time(PT) median S (range)	13.5 (12–18)	15.1 (14.3–18)	13.4 (12.7–14.1)	12.9 (12.0–13.7)	12.6 (12.1–13.1)	13.3 (12.3–14.1)
INR median (range)	1.1 (0.9–1.6)	1.23 (1.14–1.6)	1.09 (1.02–1.31)	1.02 (0.90–1.09)	0.99 (0.94–1.04)	1.04 (0.94–1.14)

**Table 2:** Characteristics of the 14 follow–up DF patients and healthy, non-dengue fever control

Variables	14 follow-up DF patients (fever duration)				Healthy (*n* = 30)	NDF (*n* = 20)
	Day 1–2	Day 3–4	Day 5–6	Day 7–8		
men/women	8/14	/	/	/	18/12	12/8
Age (yr) [range (avg)]	29 (18–50)	/	/	/	26 (19–46)	28 (21–52)
Plasma Dengue RNA median IU/m (range)	6.08 × 10^7^ (2.1 × 10^3^–1.38 × 10^8^)	3.41 × 10^7^ (231–2.39 × 10^8^)	2.86 × 10^3^ (118–1.63 × 10^7^)	349 (40.5–4.86 × 10^4^)	/	/
ALT median U/L(range)	29 (18–89)	34 (18–89)	104 (19–291)	103 (20–582)	/	/
Temperature median °C (range)	39.1 (37.8–40)	39 (37–40)	37 (36.3–37.6)	36.5 (36.2–37)	/	/
WBC median /L (range)	4.08 × 10^9^ (1.4 × 10^9^–7.9 × 10^9^)	3.5 × 10^9^ (1.4 × 10^9^–7.5 × 10^9^)	3.93 × 10^9^ (1.81 × 10^9^–5.4 × 10^9^)	5.04 × 10^9^ (2.15 × 10^9^–5.8 × 10^9^)	/	/
PLT median /L (range)	137 × 10^9^ (71 × 10^9^–217 × 10^9^)	123 × 10^9^ (67 × 10^9^–217 × 10^9^)	130 × 10^9^ (66 × 10^9^–297 × 10^9^)	226 × 10^9^ (45 × 10^9^–377 × 10^9^)	/	/
Prothrombin time (PT) median S (range)	15.9 (14–17)	15.4 (12–18)	13 (12–14)	15.6 (12–17)	/	/
INR median (range)	1.3 (1.18–1.49)	1.27 (0.92–1.55)	1.0 (0.9–1.02)	1.25 (0.88–1.55)	/	/

This study was approved by the Ethics Review Boards of Guangzhou Eighth People’s Hospital (approval number: 20160264). Written informed consent was obtained from all the study participants.

### Study design

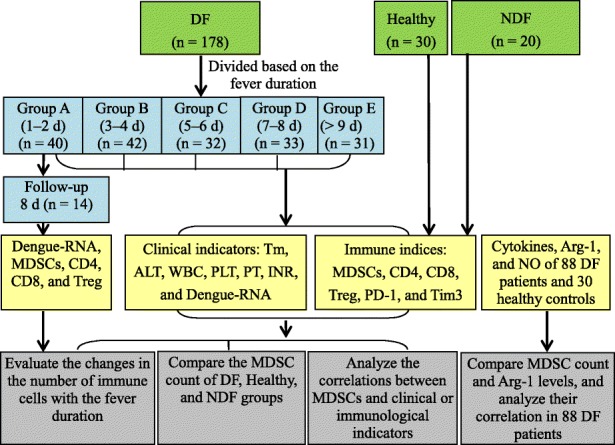



We conducted a cross-sectional study with 178 DF patients, 30 healthy donors and 20 non-dengue fever (NDF) controls. Blood samples (5 mL) were collected from all the participants, and clinical indicators, such as temperature (Tm), alanine aminotransferase (ALT), white blood cell (WBC), platelet (PLT), prothrombin time (PT), and international standardized ratio (INR), were recorded. The levels of MDSCs, CD4, CD8, Treg, PD-1, and Tim3 were evaluated by flow cytometry. Of the 178 DF patients, 88 patients were randomly selected for the assessment of their pro-inflammatory cytokine, Arg-1, and nitric oxide (NO) levels in comparison with the levels in 30 healthy controls. We aimed to assess for the differences in MDSC count among the DF patients in comparison with that of the two control groups (healthy and NDF) by evaluating the the level of MDSCs in these enrollments. Then we analyzed the correlations between MDSCs and the phenotypic features of the disease, such as the clinical indicators, and CD4, CD8, and Treg cells as well as the levels of PD-1, Tim3, Arg-1, and pro-inflammatory cytokines by using Spearman’s rank test.

Additionally, we evaluated the change in MDSC count with fever duration. DF patients were divided into 5 groups based on their fever duration, 14 patients were monitored over the course of 8 days. Changes in MDSCs count as well as other immune cells and dengue-RNA level were analyzed.

### Flow cytometric analysis of peripheral blood mononuclear cells (PBMCs)

Whole blood samples (5 mL) were collected from the patients and their PBMCs were isolated by ficoll centrifugation. The samples were immediately analyzed or cryopreserved at − 80 °C in 80% fetal calf serum, 10% RPMI–1640, and 10% dimethyl sulfoxide (DMSO) (Sigma-Aldrich, St. Louis, MO, LT: 67–68–5). The following human monoclonal antibodies were used: anti-human CD11b-PB (LT: PN B16891), anti-human CD33-PC5.5 (LT: PN A70198), anti-human HLA-DR-APC (LT: PN IM3635), anti-human CD14-ECD (LT: IM2707U), anti-human CD15-KO (LT: B01176), anti-human CD4-APC (LT:PN IM2468U), anti-human CD8-APCA700 (LT:PN A66332), anti-human Tim3-PE (LT: E17204–101), anti-human CD279-PC7 (LT: A78885), anti-human CD25-PC5.5 (LT: A79368), anti-human CD127-FITC (LT: B25366), anti-human CD19-PB (LT: A86355), and anti-human CD(16 + 56)-ECD (LT: A33098). All the antibodies were purchased from Beckman (San Diego, CA). All the experiments were performed in the biosafety laboratory.

### Evaluation of the plasma factors

The plasma arginase-1 (LT: KA3941) levels were determined using an enzyme-linked immunosorbent assay (ELISA) kit (R&D Systems, Minneapolis, MN) following the manufacturer’s instructions. The following 34 plasma factors were quantified by a Luminex-based Procarta custom 34-plex assay (eBioscience, San Diego, CA. LT: EPX340–12167-901): Eotaxin/CCL11, GM-CSF, GROα/CXCL1, IFNα, IFNγ, IL-1β, IL-1α, IL-1RA, IL-2, IL-4, IL-5, IL-6, IL-7, IL-8/CXCL8, IL-9, IL-10, IL-12 p70, IL-13, IL-15, IL-17A, IL-18, IL-21, IL-22, IL-23, IL-27, IL-31, IP-10/CXCL10, MCP-1/CCL2, MIP-1α/CCL3, MIP-1β/CCL4, RANTES/CCL5, SDF1α/CXCL12, TNFα, and TNFβ/LTA. IFN-α(LT:EPX07–10010-90) and RANTES (LT: EPX160–12176-90) levels were additionally evaluated by ELISA.

### Evaluation of NO production

NO concentrations in the plasma were measured following the manufacturer’s protocol (Biovision, Milpitas, CA, LT: K262–200). Briefly, the Greiss reagent and an equal volume of plasma (100 μL) were mixed together, then incubated for 10 min at room temperature. The absorbance at 550 nm was measured using a microplate reader (Bio-Rad, Hercules, CA). Nitrite concentrations were determined relative to a standard curve generated by the absorbance of a serially diluted sodium nitrite sample.

### Measurement of viral load

The DENV viral loads were quantified using the ABI 7300 real-time PCR system (ABI, Foster City, CA) and DENV1–4 One-Step Real-Time RT-PCR Kit (DaAn Gene, Guangzhou, Guangdong, China; Cat. #DA-N156) following the manufacturer’s instructions [13]. A 5 μL RNA sample was amplified using a program of 50 °C for 15 min, 95 °C for 15 min, followed by 40 cycles of 94 °C for 15 s and 55 °C for 45 s. Each sample was tested at least in duplicate and each experiment was performed twice and included negative and positive controls.

### Statistical analysis

All the clinical and immunological parameters were described and analyzed using non-parametric methods. Differences among groups were evaluated by the Kruskal-Wallis test followed by a modified Bonferroni test. The differences in these parameters levels in 14 DF patients among the 4 time points were evaluated by a mixed linear model. Correlations between different parameters were analyzed using Spearman’s rank test. Statistical tests were performed using GraphPad Prism version 5.0a and SPSS Statistics 17.0. *P*-values < .05 were considered significant.

## Results

### Characteristics of DF patients

The 178 DF patients had an average age of 25 years old and manifested mild fever, liver damage, and coagulation abnormalities. The average platelet level was within the normal range (125 × 10^9^–350 × 10^9^), but closer to the lower threshold. All the DF patient groups included age- and gender-matched individuals. As the fever progressed, plasma dengue viral RNA level and body temperature gradually declined (Table 1). In contrast, the ALT level gradually increased. Most of the other parameters, such as WBC, PLT, PT, and INR declined during the early phases of the fever but then returned to the normal. The 14 follow-up DF patients from whom serial samples were collected had a median age of 29 (range: 18–50). Of these, 8 (57%) patients were men. Their baseline laboratory values are shown in Table 2.

### Elevation of MDSC count in the peripheral blood of the DF patients

**Fig. 1:**
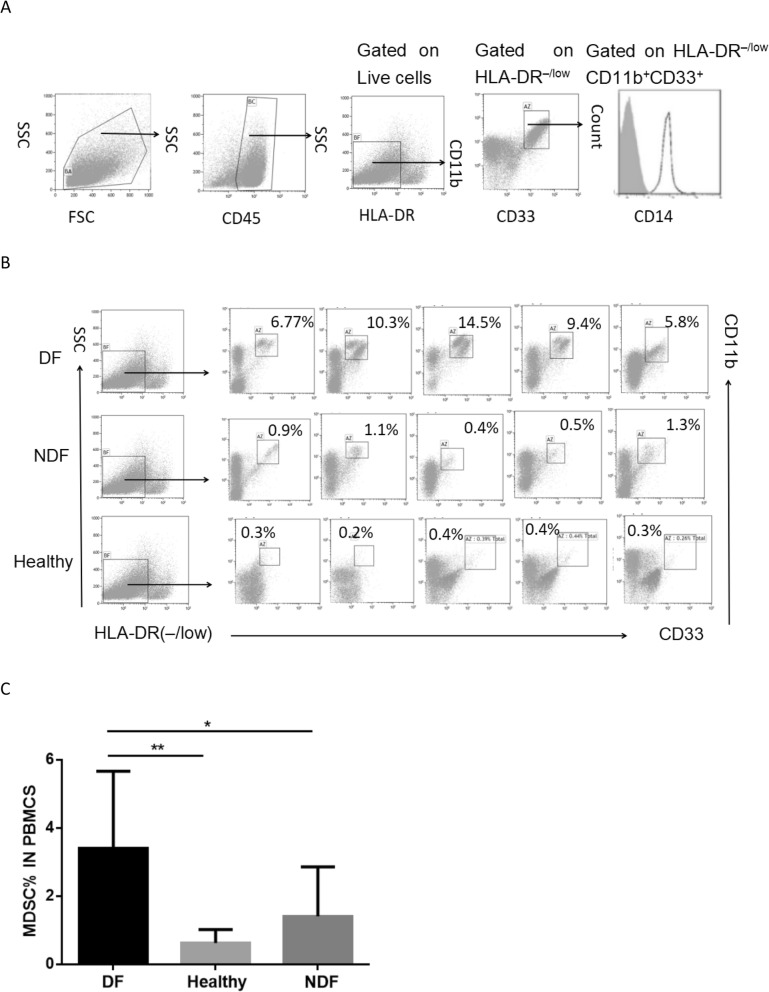
Expansion of MDSCs in the178 DF patients. Thawed 5 mL peripheral blood samples from the DF patients and healthy donors were washed with PBS, stained with specific antibodies, and analyzed by flow cytometry. **a** Gating strategy for MDSCs (HLA-DR^−/low^CD11b^+^CD33^+^) and the expression of CD14 on these cells. **b** Representative flow cytometry data from the non-severe DF patients, healthy controls, and non-dengue fever (NDF) patients. HLA-DR^−/low^ cells were first selected from live PBMCs, and the CD33^+/high^ CD11b^+^population was further gated as M-MDSCs. The boxed areas represent the percentage of M-MDSCs in PBMCs. **c** Statistical analysis of MDSC frequency in PBMCs of the DF patients (*n* = 178), healthy controls (*n* = 30) and non-dengue fever (NDF) patients (*n* = 20). Data represent median with interquartile range **-*p* < .001, *-*p* < .05

**Fig. 2:**
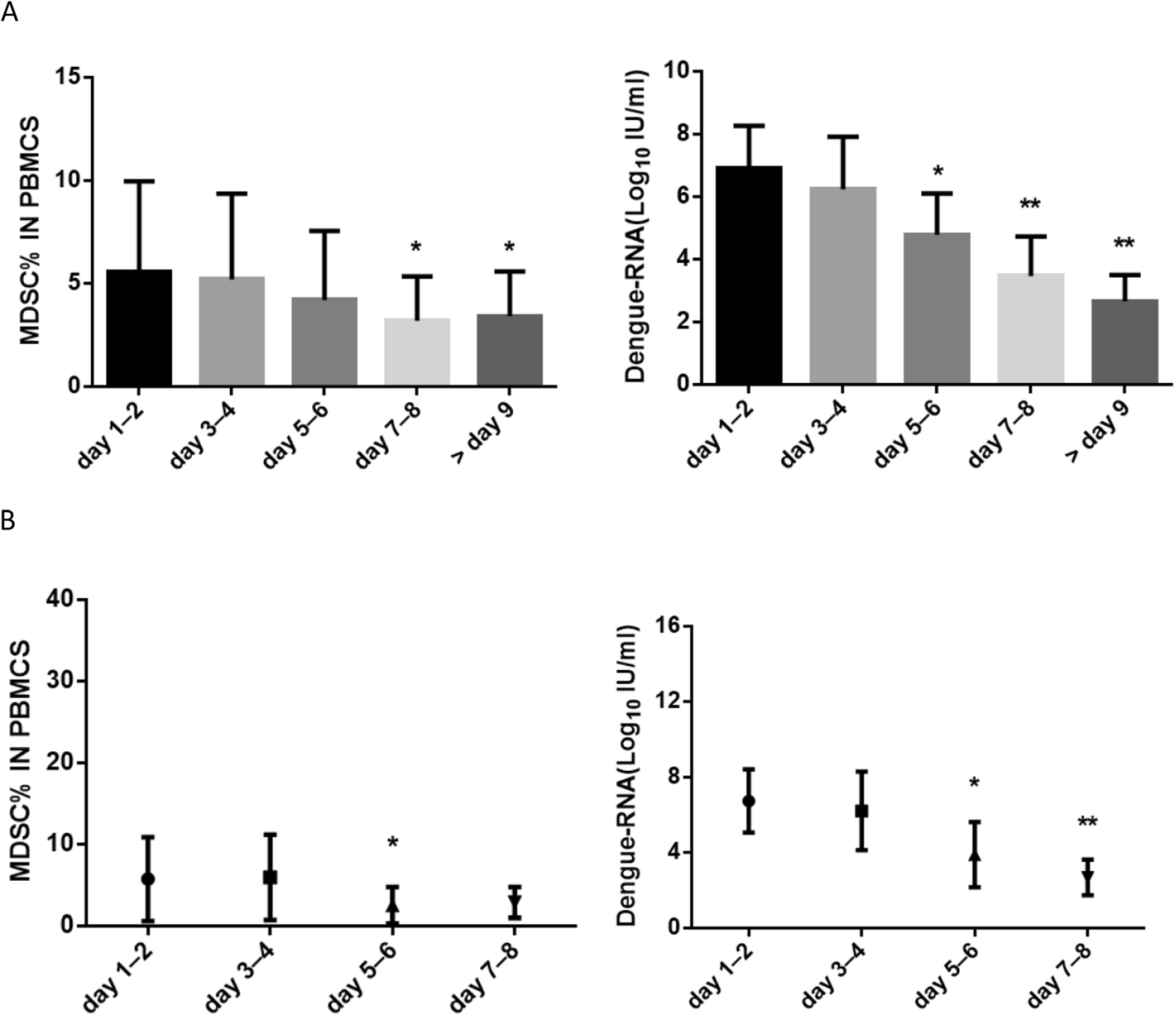
Dynamic changes in MDSCs with the increase of fever duration. Thawed peripheral blood samples from the DF patients were washed with PBS, stained with specific antibodies, and analyzed by flow cytometry. The plasma samples of the same subjects were measured for their dengue-RNA contents. Differences in (**a**) MDSC frequency and plasma dengue-RNA level with the increase in fever duration in 178 DF patients, (**b**) changes in the 14 follow-up DF patients with the increase in fever duration. Data represent the median with interquartile range. *-*p* < .05, **-*p* < .001: compared with fever duration of day 1–2

MDSCs have been shown to be of two types in humans: the monocyte MDSCs (M-MDSCs, HLA-DR^−/low^ CD11b^+^ CD33^+^ CD14^+^ CD15^−^) and granulocyte MDSCs (G-MDSCs, HLA-DR^−/low^ CD11b^+^ CD33^+^ CD14^−^ CD15^+^) [14]. In this study, high expression of CD14, but low or none expression of CD15, was detected on the MDSCs of the DF patients. Hence, the MDSCs of the DF patients were mainly CD14^+^ M-MDSCs, and their frequency in the peripheral blood of the 178 DF patients were dramatically more than in the 20 NDF controls and 30 healthy controls (Fig. 1b and c). Although they remarkably decreased in number with the increase of the fever duration and decline of the dengue viral load, they still remained more abundant than those in the two control groups even during the late phases of fever (> 9 days) (Fig. 2a). Similarly, the frequency of MDSCs in the 14 follow-up patients also remarkably decreased with the fever duration and decline of the dengue viral load but rebounded soon after 7 days of fever (Fig. 2b).

### Correlation between MDSCs and the prognostic markers and immunological markers of DF

**Fig. 3:**
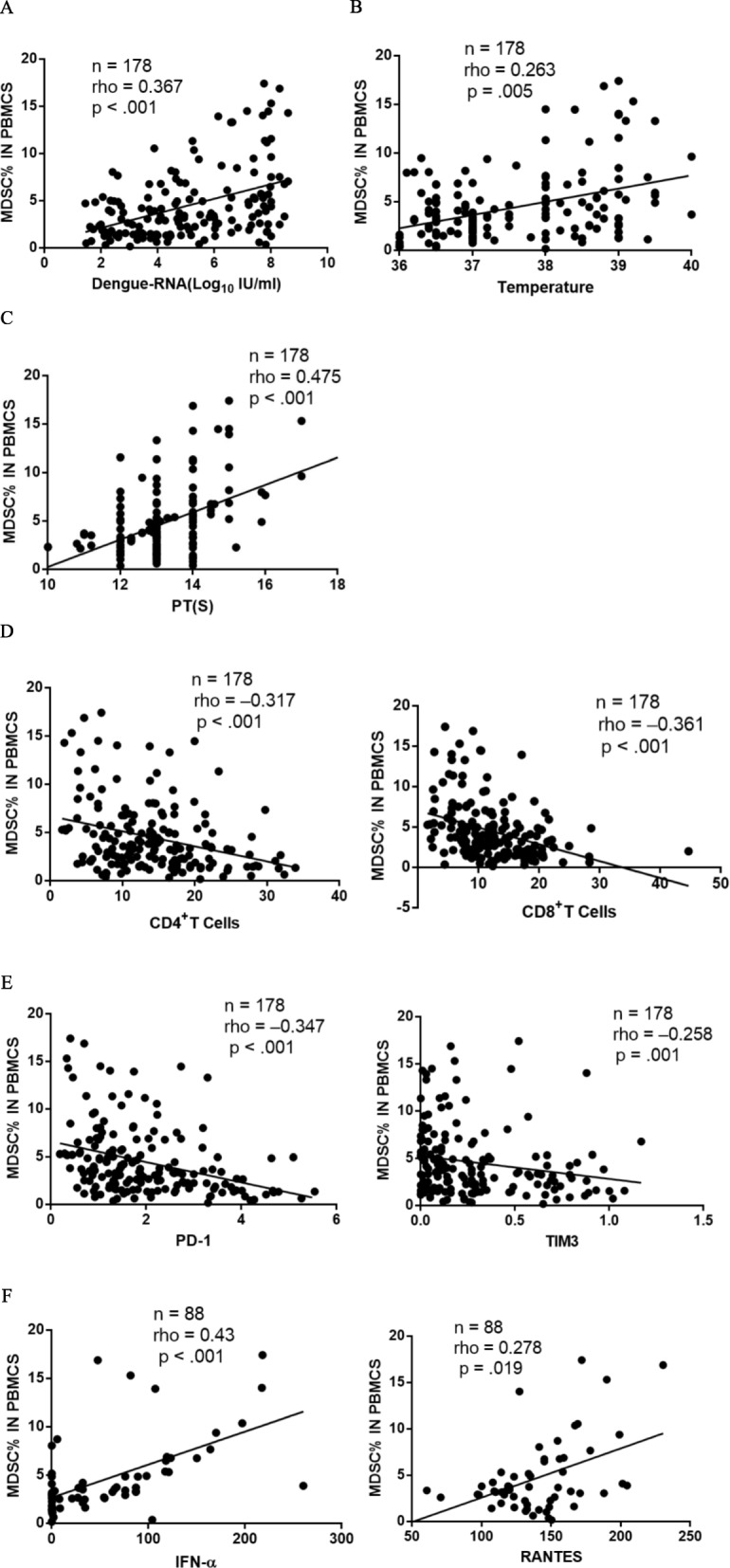
**a**–**f** Correlation between the MDSCs and DF disease markers in 178 DF patients, including (**a**) plasma dengue-RNA, (**b**) temperature of the fever patients, and (**c**) Prothrombin time (PT). The MDSC count correlated with the plasma dengue viral RNA level (*rho* = 0.367, *p* < .001), body temperature (*rho* = 0.263, *p* = .005) and prothrombin time (PT) (*rho* = 0.475, *p* < .001). Some immunological markers, including (**d**) CD4 + T and CD8 + T, (**e**) death protein 1 (PD-1) and “T cell immunoglobulin domain and mucin domain-3” (Tim3) negatively correlated with the MDSC count. Among the 178 recruited DF patients, 88 were randomly examined, and > 30 cytokines (generally involved in the cytokine storm) were tested, including interleukin, interferon, and tumor necrosis factor. Two of them significantly correlated with the MDSC count: **f** interferon-α (IFN-α) (*rho* = 0.43, *p* < .001) and “regulated upon activation normal T-cell expressed and secreted” (RANTES) (*rho* = 0.278, *p* = .019). The correlation was evaluated by Spearman’s analysis

In the DF patients, MDSC count significantly correlated with the plasma dengue viral RNA level (*rho* = 0.367, *p* < .001) (Fig. 3a), body temperature (*rho* = 0.263, *p* = .005) (Fig. 3b), PT (*rho* = 0.475, *p* < .001) and INR (*rho* = 0.451, *p* = .001) (Fig. 3c). In addition, significant negative correlation between the MDSC count and CD4^+^T (*rho* = − 0.317, *p* < .001), and CD8^+^T cell count (*rho* = − 0.361, *p* < .001) were observed (Fig. 3d). Moreover, some related immune indicators were found to be negatively correlated with the MDSC count including the PD-1 (*rho* = − 0.347, *p* < .001), and Tim3 (*rho* = − 0.258, *p* = .001) levels (Fig. 3e). Furthermore, the MDSC count significantly correlated with some cytokines involved in the cytokine storm, such as IFN-α (*rho* = 0.43, *p* < .001) and RANTES (*rho* = 0.278, *p* = .019) (Fig. 3f). Given that innate immune cells, including natural killer cells [15] and mononuclear macrophages [16] play an important role against DENV infections during the incubation phase before the specific anti-DENV responses arise, we also assessed their correlation with the MDSCs, However, no significant correlation was detected (data not shown).

### Changes in the immune cell populations with the fever duration

**Fig. 4:**
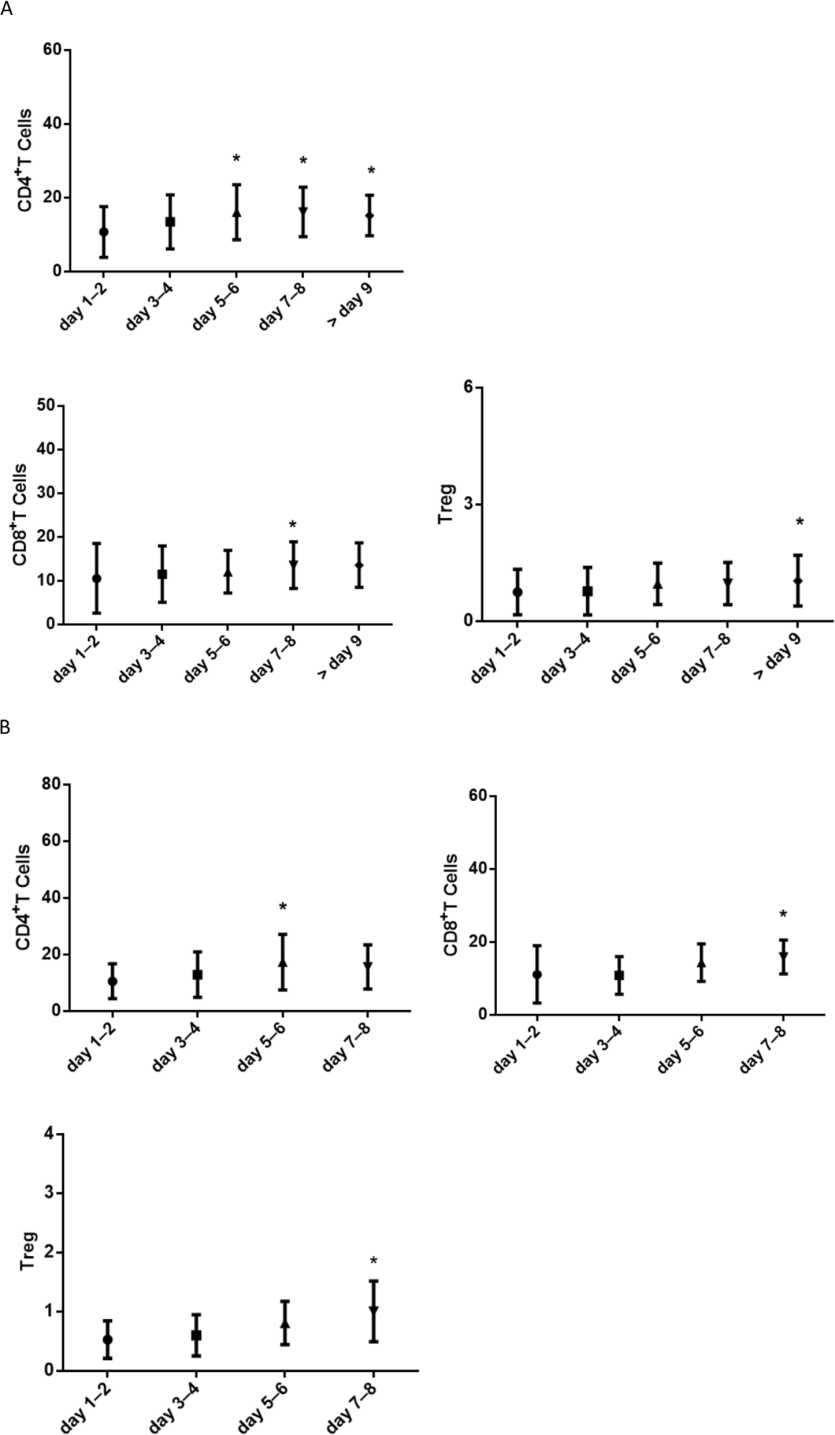
The change in immune cells at different fever duration. **a** The changes in some relevant immune cells, such as CD4^+^ T, CD8^+^ T and Treg, with the fever duration was further assessed in the 5 DF groups with different fever duration. The numbers of CD4^+^ T, CD8^+^ T, and Treg were more in patients with longer fever duration than those with shorter fever duration (data represent median with interquartile range. *-*p* < .05, compared with the fever onset duration of day1–2). **b** Similar changes in CD4 + T, CD8 + T, and Treg cells in the 14 follow-up DF patients. The numbers of CD4^+^ T, CD8^+^ T, and T reg cells gradually increased with time (data represent median with interquartile range. *-*p* < .05, compared with the fever onset duration of day1–2).

Because the MDSC count rapidly increased during the early stage of DF, changes in the numbers of some relevant immune cells, such as CD4^+^ T, CD8^+^ T, and Tregs, with fever duration were assessed for in the 5 DF groups with different fever duration. The numbers of CD4^+^ T, CD8^+^ T, and Tregs were found to be higher in patients with longer fever duration than in patients with shorter fever duration (Fig. 4a). Meanwhile, for the 14 follow-up DF patients, the numbers of CD4^+^ T, CD8^+^ T and Treg cells gradually increased with time (Fig. 4b).

### The level of arginase-1, but not NO, correlated with the MDSC count in DF patients

**Fig. 5:**
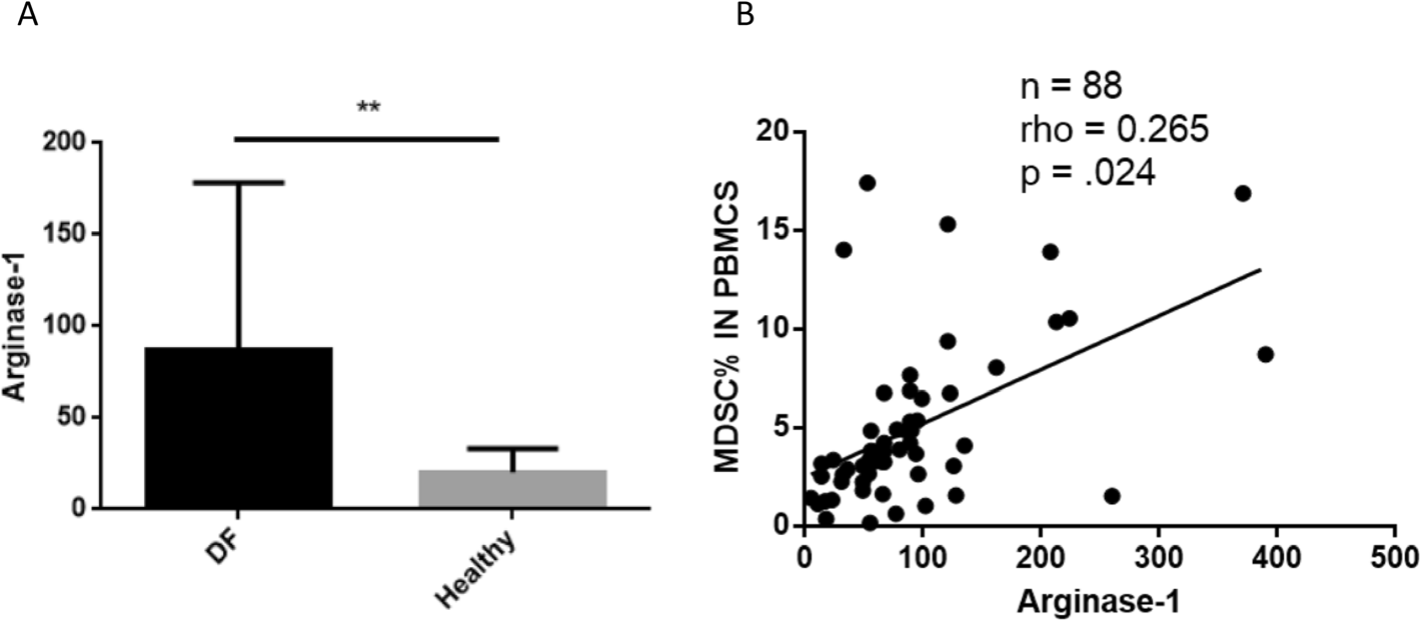
The arginase-1 levels of the 88 DF patients. The levels of the L-arginine metabolic products arginase-1 and nitric oxide (NO) were tested in the 88 of the 178 DF patients. We found (**a**) a significant increase of arginase-1 level in the PBMCs of the DF patients compared with those of the healthy controls and (**b**) a good correlation of the arginase-1 level with MDSC frequency in the 88 DF patients (*rho* = 0.265, *p* = .024). The correlation was evaluated by Spearman’s analysis and data represent the median with interquartile range. **-*p* < .001

MDSCs can suppress the functions of other immune cells, especially the T cells, by various mechanisms. It has been shown that L-arginine and its metabolic products, which serve as immune mediators, are essential for the immunosuppressive effect of MDSCs [4]. The levels of the L-arginine metabolic products arginase-1 and NO were measured in 88 of the 178 DF patients. The level of arginase-1, but not NO was higher in the DF patients than in the healthy controls (Fig. 5a) and was closely related to the MDSC count (*rho* = 0.265, *p* = .024) (Fig. 5b).

## Discussion

Although MDSCs were first studied in cancers, their involvement in non-neoplastic diseases, especially those caused by bacteria, parasites, fungi, and viruses has increasingly been investigated [9–12, 17, 18]. MDSCs may play an immunosuppressive role [17, 18] and enhance the innate immune function [19, 20], such as through activation of antigen-specific CD4+ and CD8+ T cells [4–6]. Therefore, MDSCs are expected to become a new target for the treatment of various diseases.

MDSCs can be divided into monocytic and granulocytic types, which differ in action mechanism [14]. In this study, MDSCs in the peripheral blood of the DF patients were mainly monocytic, and they were significantly more abundant than that in the controls. These results are consistent with previous reports on viral infections, such as HCV and HIV infections [10, 11], suggesting that MDSCs may mainly be involved in the pathogenesis of viral infections, including DF, in the monocytic form. It has been reported that the freezing process of PBMC may affect the abundance of some MDSC subsets in fresh blood samples [21]. To exclude the possibility that the cryopreservation process had any impact on the MDSCs expansion, we repeated our flow cytometry analysis with freshly isolated samples, and we consistently observed that the M–MDSCs were elevated in the DF patients.

We observed that the MDSCs level significantly decreased with the increase of the fever duration, and correlated with several biochemical parameters in the DF patients, including DENV load, body temperature, PT, and INR, indicating that MDSCs may be associated with disease progression. Interestingly, MDSCs were significantly more abundant in DF patients than in NDF controls, although MDSCs are known to be elevated in almost all inflammatory diseases. These findings may suggest that MDSCs play a more significant role in the pathogenesis of dengue fever than other infectious diseases.

The strong correlation between MDSCs and DENV load suggests that DENV itself may drive MDSC expansion. This possibility is consistent with observations regarding other viruses, such as HCV [10] and HIV [11]. In addition to the direct effect of DENV, other factors may drive MDSC expansion. DENV has been shown to infect a wide range of cells to produce pro-inflammatory cytokines required for MDSC development and generation [22]. We found a significant correlation of MDSC count with IFN-α and RANTES levels. The former may play an important role in the primary defense against DENV infection [23], while the latter may contribute to the infiltration of the immune cells [24]. Taken together, these results indicate that MDSCs may be involved in the “cytokine storm” during DF, and play paradoxical roles in the progression of DF.

The sustained elevation in MDSC count in DF patients with completely undetectable plasma levels of DENV RNA raises the possibility that M-MDSCs also play a role during the late stage of the disease. Tregs (CD4^+^CD25^+^FoxP3^+^) are known to inhibit effector T cell proliferation and functions [25]. Previous studies have confirmed that MDSCs can promote the induction of Treg cells [26]. In the present study, although we didn’t find any correlation between the MDSC and Treg cell numbers, the abundance of MDSCs may indirectly cause an immunosuppressive effect by promoting Treg proliferation during the advanced stages of DF. However, the role of MDSCs on Treg cell development remains to be clarified. In contrast, the CD4^+^ T and CD8^+^ T cell numbers increased with the fever duration, in negative correlation with the MDSC count, indicating that MDSCs might suppress T-cell function. These observations are consistent with the results of previous studies [4, 5].

L-arginine and its metabolic products, such as arginase-1, nitric oxide (NO), and reactive oxygen species (ROS), which serve as immune mediators, are essential for the suppressive effect of MDSCs [4, 27]. As expected, our study found that arginase-1 was upregulated in DF patients and might participate in the inhibitory functions of MDSCs. In this study, we did not observe a noticeable difference in the NO level in DF patients than in controls. We were unable to detect the factors related to ROS due to the limited number of cells collected.

MDSC expansion is a common response to most inflammatory processes, and the functions of MDSCs are highly dependent on the inflammatory environment. Although clinical treatments have been developed to downregulate MDSCs in cancers so that antineoplastic responses are improved [4], recent studies suggest that the MDSCs expansion in acute inflammatory processes, such as burns and sepsis may play a beneficial role by regulating immune surveillance and innate immune responses [20, 22]. In DF, MDSCs may also protect hosts by decreasing the magnitude of the inflammatory response.

In summary, our results present new insights into the possible functions of MDSCs and their role in DF patients. Our study may be useful for the development of immunotherapy against DF by targeting the MDSCs.

## Conclusions

The findings of this study suggest that MDSCs are elevated in DF, and reveal a significant correlation between MDSCs and the clinical indicators of DF. Although the functions of MDSCs in DF were not examined, we found that the level of arginase-1 was increased. Which is essential for MDSC-mediated suppressive functions. Thus, Our study demonstrates that MDSCs may be involved in the immunological reaction to DF.

## Data Availability

Data cannot be made publicly available due to ethical restrictions imposed by the Ethical Committee of Guangzhou Eighth People’s hospital on human rights related to research. Request for data can be sent to chief Ni, head of the Ethical Committee.

## Abbreviations

AIDS: acquired immune deficiency syndrome
ALT: alanine aminotransferase
DENV: dengue virus
DF: dengue fever
DMSO: dimethyl sulfoxide
ELISA: enzyme-linked immunosorbent assay
IFN-α: interferon-α
INR: international standardized ratio
MDSCs: Myeloid-derived suppressor cells
NDF: non-dengue fever
NO: nitric oxide
NS1: nonstructural protein 1
PBMCs: peripheral blood mononuclear cells
PD-1: programmed cell death protein 1
PLT: platelet
PT: prothrombin time
RANTES: regulated upon activation normal T-cell expressed and secreted
ROS: reactive oxygen species.
RT-PCR: reverse transcriptionpolymerase chain reaction
Tim3: T cell immunoglobulin domain and mucin domain-3
WBC: white blood cell
